# New non-ureolytic heterotrophic microbial induced carbonate precipitation for suppression of sand dune wind erosion

**DOI:** 10.1038/s41598-023-33070-w

**Published:** 2023-04-10

**Authors:** Mohammad Hemayati, Ehsan Nikooee, Ghassem Habibagahi, Ali Niazi, Sayed Fakhreddin Afzali

**Affiliations:** 1 Department of Civil and Environmental Engineering, School of Engineering, Shiraz University, Zand Street, Shiraz, 71348-51156 Iran; 2 Institute of Biotechnology, Shiraz University, Shiraz, Iran; 3 Department of Natural Resource and Environmental Engineering, Shiraz University, Shiraz, Iran

**Keywords:** Biogeochemistry, Environmental sciences, Civil engineering

## Abstract

The detrimental effects of sand storms on agriculture, human health, transportation network, and infrastructures pose serious threats in many countries worldwide. Hence, wind erosion is considered a global challenge. An environmental-friendly method to suppress wind erosion is to employ microbially induced carbonate precipitation (MICP). However, the by-products of ureolysis-based MICP, such as ammonia, are not favorable when produced in large volumes. This study introduces two calcium formate-bacteria compositions for non-ureolytic MICP and comprehensively compares their performance with two calcium acetate-bacteria compositions, all of which do not produce ammonia. The considered bacteria are* Bacillus subtilis* and* Bacillus amyloliquefaciens*. First, the optimized values of factors controlling CaCO_3_ production were determined. Then, wind tunnel tests were performed on sand dune samples treated with the optimized compositions, where wind erosion resistance, threshold detachment velocity, and sand bombardment resistance were measured. An optical microscope, scanning electron microscope (SEM), and X-ray diffraction analysis were employed to evaluate the CaCO_3_ polymorph. Calcium formate-based compositions performed much better than the acetate-based compositions in producing CaCO_3_. Moreover,* B. subtilis* produced more CaCO_3_ than* B. amyloliquefaciens*. SEM micrographs clearly illustrated precipitation-induced active and inactive bounds and imprints of bacteria on CaCO_3_. All compositions considerably reduced wind erosion.

## Introduction

Wind erosion has always been considered one of the significant problems in arid and semi-arid regions, such as the southwest USA, western China, Saharan Africa, and most of the Middle East^1^. Scant rainfall patterns of arid and ultra-arid climate conditions transform most of these regions into deserts, sand dunes, and uncultivated lands. The persistent wind-induced erosion of sand dunes poses an environmental threat to infrastructures such as transportation networks, agricultural land, and industrial sites making life difficult and urban developments in such regions costly^2–4^. It is important to note that wind erosion affects not only its region of origin but also brings about health and economic problems for far settlements due to its induced wind-borne particles carried to regions far from the source^5,6^.

Controlling wind erosion remains a global challenge. Various soil stabilizing methods have been employed to control wind erosion. These methods include the application of materials such as water^7^, petroleum mulches^8^, biopolymers^5^, microbially induced carbonate precipitation (MICP)^9–12^ and also, enzyme-induced carbonate precipitation (EICP)^1^. Soil wetting is the standard method for dust suppression in the field. However, its rapid evaporation leaves this method with limited effectiveness in arid and semi-arid regions^1^. The application of petroleum mulch compounds increases sand cohesion and inter-particle friction. Their cohesive essence binds the sand particles together; however, petroleum mulches bring about other issues; their dark color increases heat absorption and perishes plants and microorganisms. Their odor and vapor cause respiratory problems, and most notably, their excessive price is another handicap. Biopolymers are among the recently suggested environmental-friendly methods for mitigating wind erosion; they are extracted from natural resources such as plants, animals, and bacteria. Xanthan gum, Guar gum, Chitosan, and Gellan gum are the most typically used biopolymers in engineering^5^. However, water-soluble biopolymers may lose their strength and be washed away from the soil when exposed to water^13,14^. EICP has been demonstrated to be an effective method for dust suppression in various applications, including unpaved roads, mine tailings, and construction sites. In spite of its promising results, there are also some potential disadvantages to consider such as cost and lack of nucleation sites which expedite the formation and precipitation of CaCO_3_ crystals^15,16^.

MICP was first referred to in the late nineteenth century in the studies of Murray and Irvine (1890) and Steinmann (1901), together with urea decomposition by the marine microbiota^17^. MICP is a natural biological process associated with various microbial activities and chemical processes, during which calcium carbonate precipitation occurs as a result of microbial metabolic products such that carbonate ions react with calcium ions in the environment^18,19^. MICP involving nitrogen cycle by the degradation of urea (ureolytic MICP) is the most common type of microbial-induced carbonate precipitation, in which the urease enzyme generated by the bacteria catalyzes the hydrolysis of urea^20–27^, described by the following reactions:

1 $${\text{CO}}\left( {{\text{NH}}_{2} } \right)_{2} + 2{\text{H}}_{2} {\text{O}}\xrightarrow{{{\text{Urease enzyme}}\;\left( {{\text{Ureolytic bacteria}}} \right)}}2{\text{NH}}_{3} + {\text{H}}_{2} {\text{CO}}_{3}$$

2 $${\text{NH}}_{3} + {\text{H}}_{2} {\text{O}} \to {\text{NH}}_{4}^{ + } + {\text{OH}}^{ - }$$

3 $${\text{H}}_{2} {\text{CO}}_{3} \leftrightarrow {\text{HCO}}_{3}^{ - } + {\text{H}}^{ + } \to 2{\text{H}}^{ + } + {\text{CO}}_{3}^{2 - }$$

4 $${\text{Ca}}^{2 + } + {\text{CO}}_{3}^{2 - } \to {\text{CaCO}}_{3}$$

In MICP involving the carbon cycle by oxidation of organic salts (non-ureolytic MICP), heterotrophic bacteria utilize organic salts like acetate, lactate, citrate, succinate, oxalate, malate, and glyoxylate as sources of energy to produce carbonate minerals^28^. The chemical reactions for the formation of calcium carbonate in the presence of calcium lactate as a source of carbon and calcium ions are given in Eq. (5).

5 $${\text{CaC}}_{6} {\text{H}}_{10} {\text{O}}_{6} + {\text{O}}_{2} \to {\text{CaCO}}_{3} + {\text{H}}_{2} {\text{O}} + {\text{CO}}_{2}$$

In the MICP process, bacterial cells provide nucleation sites that are of particular importance for calcium carbonate precipitation; the surfaces of bacterial cells are negatively charged, and act as adsorbents of divalent cations such as calcium ions. By adsorbing calcium ions on the bacterial cell, with a sufficient concentration of carbonate ions, the reaction of calcium cation and carbonate anion is performed, and calcium carbonate is precipitated on the surface of bacteria^29,30^. The process could be summarized as follows^31,32^:

6 $${\text{Ca}}^{2 + } + {\text{Bacteria cell}} \to {\text{Cell - Ca}}^{2 + }$$

7 $${\text{Cell - Ca}}^{2 + } + {\text{CO}}_{3}^{2 - } \to {\text{Cell - CaCO}}_{3}$$

Crystals of biologically produced CaCO_3_ can be divided into three types: calcite, vaterite, and aragonite. Among these, calcite and vaterite are the most common bacterial-induced CaCO_3_ polymorphs^33,34^. Calcite is the most thermodynamically stable polymorph of CaCO_3_^35^. While vaterite has been reported to be of metastable nature, it is eventually transformed to calcite^36,37^. Vaterite has the highest density among these crystals. It is a hexagonal crystal whose pore-filling capability is better than that of other CaCO_3_ crystals because of its large volume^38^. Both ureolytic and non-ureolytic MICP can result in vaterite precipitation^13,39–41^.

Having reviewed the recent literature on MICP, the following gaps can be noticed:

Although MICP has shown promising potential for stabilizing problematic soils as well as soils prone to wind erosion^42–48^, one by-product of urea hydrolysis is ammonia, which may cause mild to severe health issues depending on the level of exposure^49^. This side effect makes the use of this particular technique of the MICP controversial, particularly when vast areas (as in dust suppression application) should be treated. Besides, the ammonia odor is hard to tolerate when the process is performed at a high application rate and in large volumes, which may influence its practical applicability. Although recent studies have suggested reducing the amount of ammonium ions by conversion of them to other products such as struvite, such methods do not result in total ammonium ions removal^50^. Alternative solutions which involve no-ammonium ion production are therefore still required to be investigated. The use of non-ureolytic pathway for MICP can provide a potential solution, which has rarely been studied for wind erosion mitigation. Fattahi et al. investigated non-ureolytic MICP by means of calcium acetate and bacillus megaterium^41^. While Mohebbi et al. employed calcium acetate and bacillus amyloliquefaciens^9^. However, their study did not include a comparison with other calcium sources and heterotrophic bacteria, which could eventually result in higher wind erosion resistance. The literature also lacks a comparison between the non-ureolytic and ureolytic pathways for wind erosion mitigation.

Furthermore, most wind erosion and dust control studies have been performed on soil samples with flat surfaces^1,51–53^. However, in nature, flat surfaces are rare to find compared to hills and depressions. That is why sand dunes are the most common scenery in desert areas.

To alleviate the above-mentioned shortcomings, the current study aims to introduce a new set of bacteria-substances that do not produce ammonia. For this purpose, non-ureolytic MICP pathway has been considered. The performance of two calcium sources (calcium formate and calcium acetate) has been examined. Carbonate precipitation by means of two calcium source-bacteria compositions, namely, calcium formate-bacillus subtilis, and calcium formate-bacillus amyloliquefaciens has not been studied in the previous studies to the authors’ knowledge. The selection of these bacteria has been based on the enzymes they produce, such that they can catalyze the oxidation of both calcium formate and calcium acetate, thereby, resulting in microbial carbonate precipitation. A careful experimental study is designed to find optimum factors such as pH, bacteria, and calcium source type as well as their concentration, bacterial to calcium source solution ratio, and curing time. Finally, the efficacy of this set of bacteria-substances in suppression of wind erosion by means of calcium carbonate precipitation is investigated using a series of wind tunnel tests on sand dunes to determine the wind erosion amount, threshold detachment velocity as well as resistance against sand bombardment accompanied by penetrometer measurements and microfabric studies (e.g., X-ray diffraction (XRD) analysis and scanning electron microscope (SEM)).

## Materials and methods

### Materials

Calcium ions and carbonate ions are required to produce calcium carbonate. Calcium ions can be obtained from various calcium sources, such as calcium chloride, calcium hydroxide, and non-fat powder milk^54,55^. Different processes can be utilized to generate carbonate ions microbially, such as hydrolysis of urea and aerobic or anoxic oxidation of organics^56^. In the current study, carbonate ions were produced from formate and acetate oxidation reactions. Furthermore, we used calcium salts of formate and acetate to produce pure calcium carbonate, and thereby, resulting in only CO_2_ and H_2_O as a by-product. In this process, merely one substance acts as the calcium and carbonate source and does not produce ammonia. Such characteristics make the considered calcium sources and the process of carbonate production highly desirable.

The reactions pertinent to calcium carbonate production from calcium formate and calcium acetate are presented by Eqs. (7)–(14). Equations (7)–(11) indicate that calcium formate dissolves in water, producing formic acid or formate. Consequently, this solution is a source of free calcium and hydroxide ions (Eqs. 8, 9). Whereas, through formate oxidation, the carbon atoms of formate are converted to carbon dioxide (Eq. 10). Eventually, calcium carbonate is produced (Eqs. 11, 12).

8 $${\text{Ca}}\left( {{\text{HCOO}}} \right)_{2} + {\text{H}}_{2} {\text{O}} \to {\text{HCOOH}} + {\text{Ca}}^{2 + } + {\text{OH}}^{ - }$$

9 $${\text{HCOOH}} \to {\text{HCOO}}^{ - } + {\text{H}}_{2}$$

10 $${\text{HCOO}}^{ - } + {\text{O}}_{2} \to {\text{CO}}_{2} + {\text{H}}_{2} {\text{O}}$$

11 $${\text{CO}}_{2} + {\text{H}}_{2} {\text{O}} \leftrightarrow {\text{H}}_{2} {\text{CO}}_{3} \leftrightarrow {\text{H}}^{ + } + {\text{HCO}}_{3}^{ - } \leftrightarrow {\text{H}}^{ + } + {\text{CO}}_{3}^{2 - }$$

12 $${\text{Ca}}^{2 + } + {\text{CO}}_{3}^{2 - } \to {\text{CaCO}}_{3}$$

Similarly, calcium carbonate is produced via calcium acetate (Eqs. 13–15) with the difference that it produces acetic acid or acetate instead of formic acid.

13 $${\text{Ca}}\left( {{\text{CH}}_{3} {\text{COO}}} \right)_{2} + {\text{H}}_{2} {\text{O}} \to {\text{CH}}_{3} {\text{COOH}} + {\text{ Ca}}^{2 + } + {\text{OH}}^{ - }$$

14 $${\text{CH}}_{3} {\text{COOH}} \to {\text{CH}}_{3} {\text{COO}}^{ - } + {\text{H}}_{2}$$

15 $${\text{CH}}_{3} {\text{COO}}^{ - } + {\text{O}}_{2} \to {\text{CO}}_{2} + {\text{H}}_{2} {\text{O}}$$

Oxidation of both acetate and formate at the ambient temperature cannot be instigated without the presence of enzymes. FDH (Formate dehydrogenase) and CoA (Coenzyme A) are enzymes that catalyze the formate and acetate oxidations, respectively, to produce carbon dioxide (Eqs. 16,17)^57–59^. Various bacteria produce these enzymes, and in this study, heterotrophic bacteria, namely, Bacillus subtilis (PTCC no. 1204 (Persian Type Culture Collection), specified also as NCIMB no. 13061 (International Depository Authority for bacteria, yeasts, bacteriophages, plasmids, plant seeds and plant cell tissue cultures)) and Bacillus amyloliquefaciens (PTCC no. 1732, NCIMB no. 12077) were used. These bacteria were cultivated in the medium with the ingredient of peptone from meat (5 g/L) and meat extract (3 g/L), which is called Nutrient Broth (NB) (105443 Merck).

16 $${\text{HCOO}}^{ - } + {\text{O}}_{2} \xrightarrow{{{\text{FDH Enzyme }}\left( {{\text{Heterotrophic Bacteria}}} \right)}}{\text{CO}}_{2} + {\text{H}}_{2} {\text{O}}$$

17 $${\text{CH}}_{3} {\text{COO}}^{ - } + {\text{O}}_{2} \xrightarrow{{{\text{CoA }}\left( {{\text{Heterotrophic Bacteria}}} \right)}}{\text{CO}}_{2} + {\text{H}}_{2} {\text{O}}$$

Therefore, two calcium sources and two types of bacteria were used to make four compositions to induce calcium carbonate precipitation: calcium formate and bacillus subtilis (FS), calcium formate and bacillus amyloliquefaciens (FA), calcium acetate and bacillus subtilis (AS), and calcium acetate and bacillus amyloliquefaciens (AA).

### Experimental program

In the first part of the experimental program, tests were carried out to determine the optimized combinations that would lead to the maximum amount of calcium carbonate production. Due to the presence of calcium carbonate in the soil samples and in order to have accurate measurements of the produced CaCO_3_, for different combinations, a set of initial assessment tests were designed and carried out on the mixture of culture medium bacterial solution and calcium source solution. The obtained optimized factors (calcium source concentration, curing time, bacterial concentration measured in terms of solution optical density (OD), the ratio of calcium source to the bacterial solution, and pH) for each calcium source-bacteria composition (FS, FA, AS, and AA), defined above, were then used in the wind tunnel tests on treated sand dunes, described in the subsequent sections.

#### Optimization of CaCO_3_

A Series of 150 experiments for each composition was performed to investigate CaCO_3_ precipitation by assessing the influence of various factors, that is, calcium source concentration, curing time, bacterial OD, the ratio of calcium source to the bacterial solution, and pH during aerobic oxidation of organics (Table 1). The selection of pH range for the optimization process was made based on the growth curve of Bacillus subtilis and Bacillus amyloliquefaciens, so that a faster growth rate could be achieved. It has been further clarified in the “Result” section.

**Table 1:** List of the optimization factors of each composition.

Factors	Level 1	Level 2	Level 3
OD	0.5	1	1.5
Concentration of calcium source (g/L)	10	30	50
Ratio of calcium source to bacterial solution	0.5	1	2
pH of Bacillus subtilis culture medium	7	8	9
pH of Bacillus amyloliquefaciens culture medium	6	7	8
Curing time (days)	3	6	9

**Figure 1:**
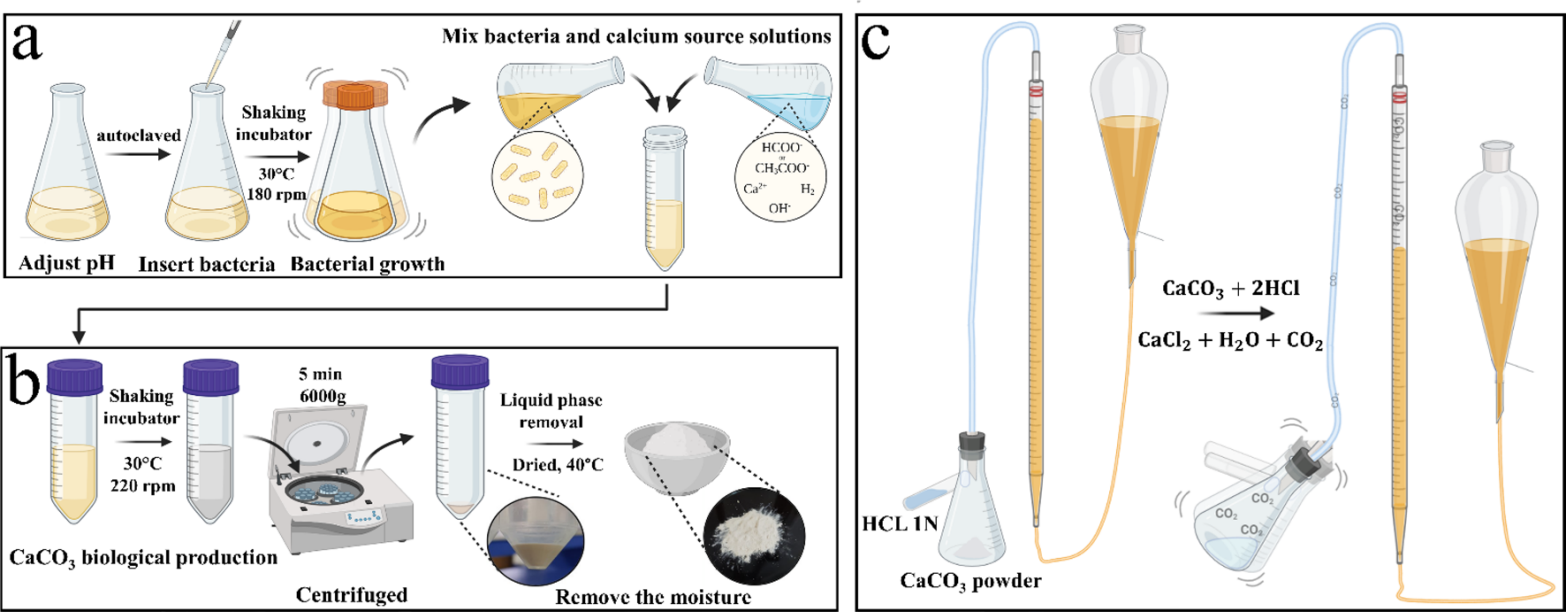
Schematic presentation of CaCO_3_ production optimization stages.

The following procedure was adopted to prepare samples for the optimization phase. MICP solution was prepared by first adjusting the initial pH of the culture medium and autoclaving at 121 °C for 15 min. Next, inoculation of the strains was performed in laminar airflow, and they were kept in a shaking incubator at 30 °C and 180 rpm. As soon as the OD of the bacteria reached the desired level, it was mixed with the calcium source solution in the intended ratio (Fig. 1a). The MICP solution was allowed to react and cured in a shaking incubator at 220 rpm and 30 °C for the targeted curing time. Precipitated CaCO_3_ was separated after centrifugation for 5 min at 6000 *g*, then dried at 40 °C to prepare the specimen for calcimeter test (Fig. 1b). A Bernard calcimeter was then used to measure CaCO_3_ precipitation, in which, as a result of the reaction of CaCO_3_ powder with 1.0 N HCl (ASTM-D4373-02), CO_2_ is produced, and the volume of this gas is a measure of the amount of CaCO_3_ (Fig. 1c). In order to convert CO_2_ volume to CaCO_3_ content, a calibration curve was obtained by washing pure CaCO_3_ powder with HCl 1N and plotting it against emitted CO_2_. The morphology and purity of the precipitated CaCO_3_ powder were examined using SEM imaging and XRD analysis. In order to inspect the process of calcium carbonate production around bacteria, the phase of produced calcium carbonate, as well as the activity of bacteria, an optical microscope with a magnification of 1000 was employed.

#### Wind erosion of sand dunes and surface strength measurements

**Figure 2:**
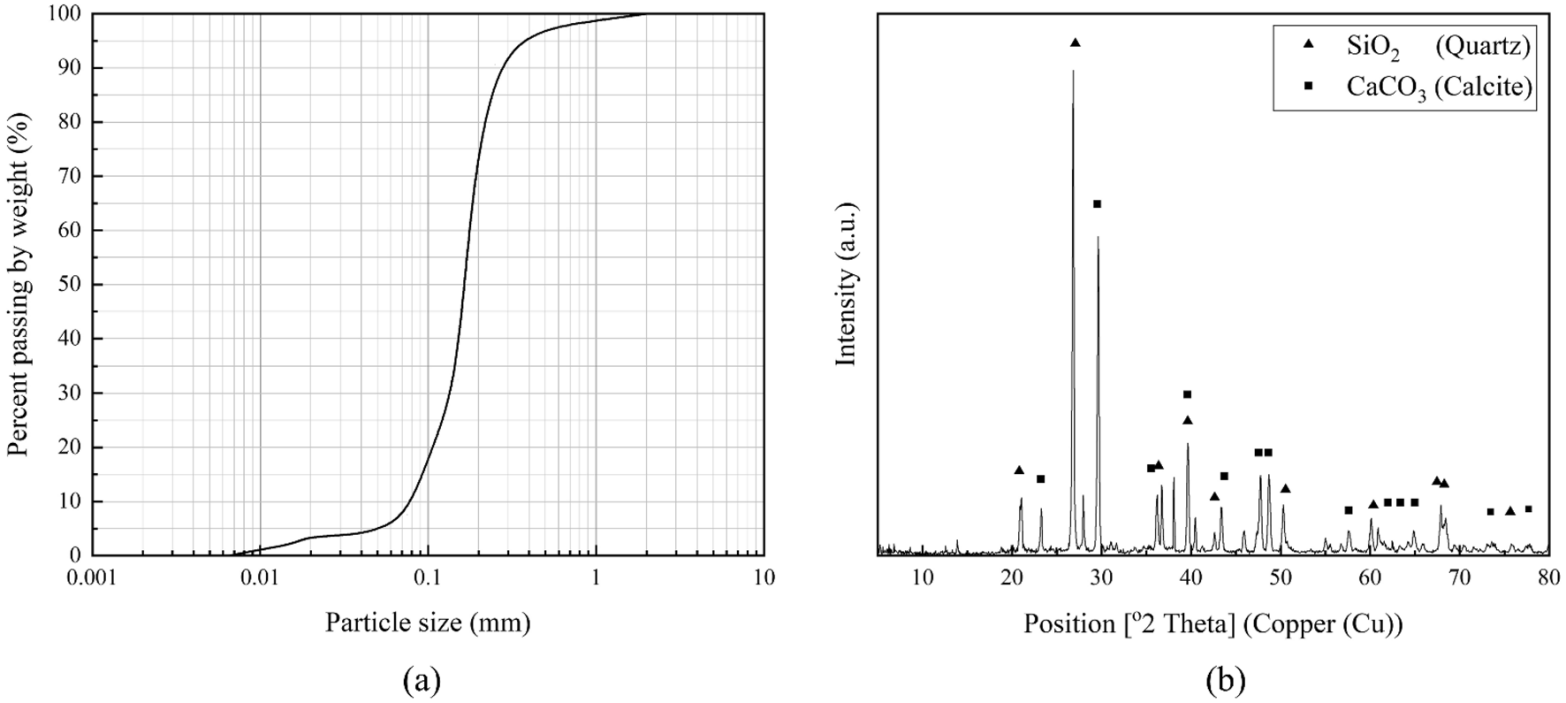
(**a**) Dejgah soil particle size distribution, (**b**) XRD spectra of the Dejgah soil.

Dejgah catchment is a region with known severe erosion in southwestern Fars in Iran, from which soil samples susceptible to wind erosion were obtained. The samples were collected from the topsoil layer of this region for investigation. Index tests on the soil samples indicated that the soil is a poorly graded sand with silt, classified as SP-SM based on the Unified Soil Classification System (Fig. 2a). XRD analysis revealed that the Dejgah soil is mainly composed of calcite and quartz (Fig. 2b). In addition, EDX analysis showed that other elements such as Al, K, and Fe are also present in lower proportions.

To prepare laboratory sand dunes for wind erosion tests, the soil was pulverized through a funnel of a diameter of 10 mm and from a height of 170 mm on a rigid surface resulting in sand dunes typically 60 mm in height and 210 mm in diameter. In nature, sand dunes are formed at their lowest density through an aeolian process. Similarly, the samples prepared using the above-mentioned procedure had the lowest relative density for the sand, γ = 14.14 kN/m^3^, forming a sand cone piled on a horizontal surface with its angle of repose roughly equal to 29.7°.

**Figure 3:**
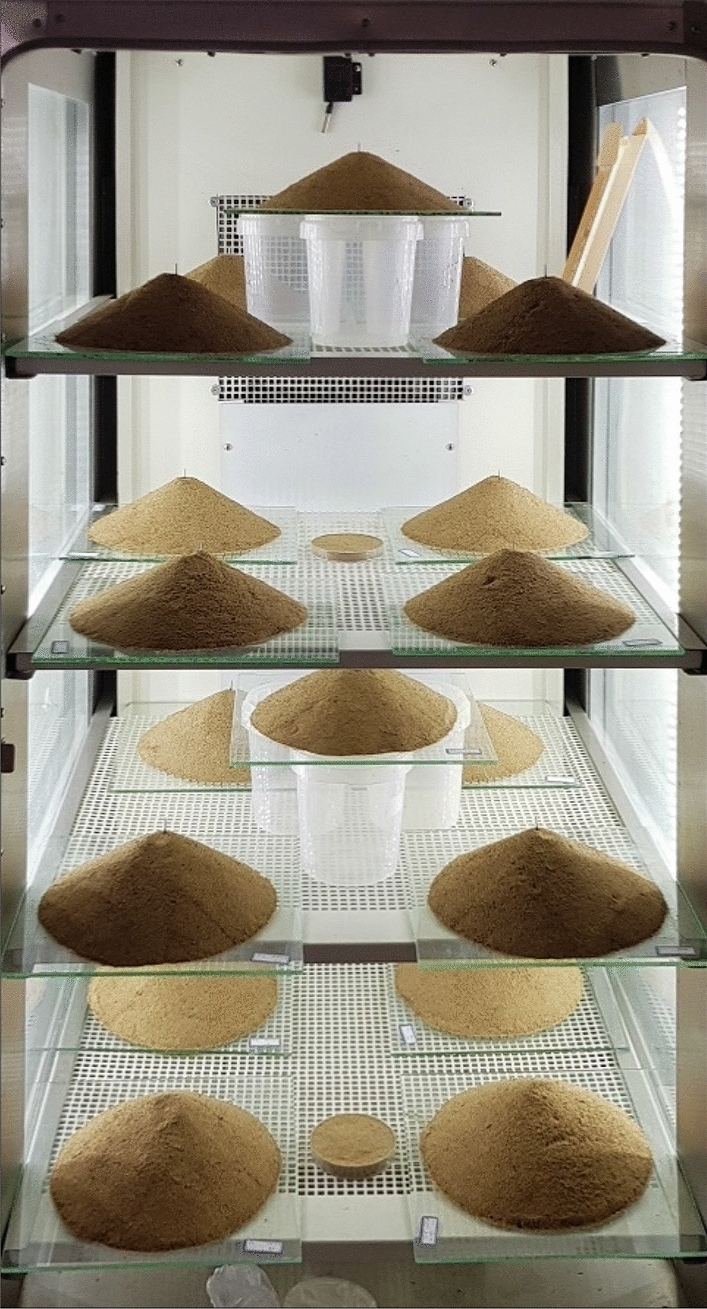
Treated sand dunes in an incubator (kept at 30 °C).

The optimum MICP solution obtained from the previous section was sprayed on the lateral surface of the sand dunes at 1, 2, and 3 L.m^-2^ application rates and the samples were then stored in an incubator at 30 °C (Fig. 3) for a period of 9 days (i.e., the optimum curing time) before removing for tests in the wind tunnel.

Four samples were prepared for each treatment scenario, one for the calcium carbonate content measurement as well as surface strength measurement by penetrometer, and the remaining three samples were used for the erosion tests at three different speeds. In the wind tunnel tests, the amounts of erosion at different wind speeds were determined, and a diagram showing erosion amount versus wind velocity was then used to find the threshold detachment velocity for each treated sample. In addition to the wind erosion testing, the treated samples were also exposed to sand bombardment (i.e., saltation experiments). For this purpose, two additional samples were prepared at an application rate of 2 and 3 L m^−2^. The sand bombardment tests were carried out for a period of 15 min and at a flux of 120 g m^−1^_,_ which is within the range of values selected in the previous studies^60–62^. The horizontal distance between the abrader nozzle and the toe of the dune was 800 mm, and it was located at a distance of 100 mm above the tunnel floor. This location had been set such that almost all the saltator sand grains could hit the dune.

**Figure 4:**
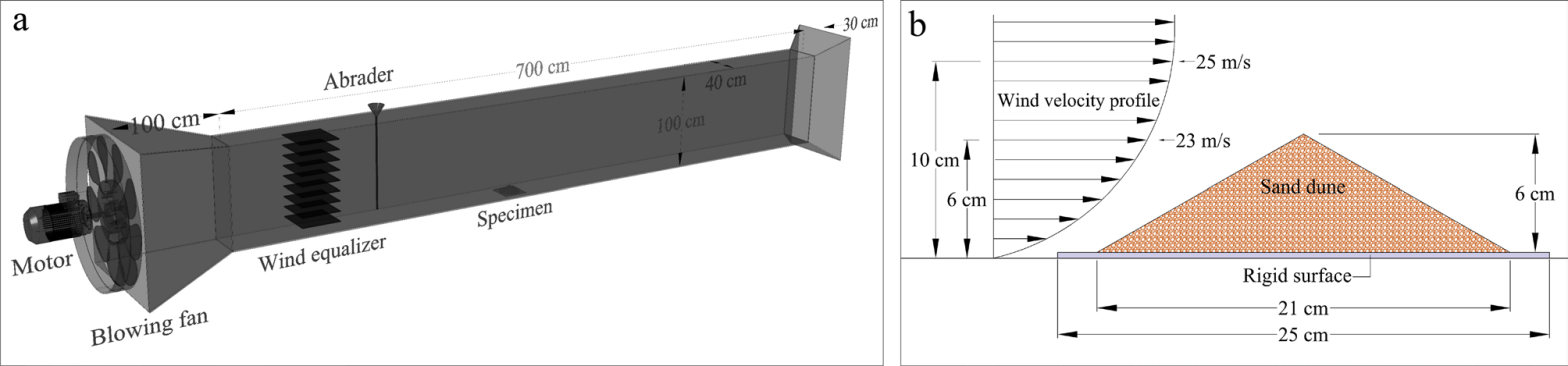
(**a**) Schematic of the wind tunnel, (**b**) Wind velocity profile over the sand dune samples.

Wind tunnel experiments were conducted in an open circuit wind tunnel 8 m long, 0.4 m wide, and 1 m in height (Fig. 4a). The tunnel was made of galvanized steel sheets and could generate wind speed up to 25 m s^−1^. Moreover, an inverter drive was employed to adjust the frequency of the blowing fan while the frequency was gradually augmented to generate the target wind speed. Figure 4b illustrates the schematic of sand dunes subjected to wind erosion and the measured wind velocity profile in the tunnel.

Finally, in order to compare the results of non-ureolytic MICP compositions proposed in this study with control ureolytic MICP tests, sand dune samples were also prepared and treated with the biological solution containing urea, calcium chloride, and Sporosarcina Pasteurii (Since Sporosarcina pasteurii has pronounced urease enzyme production aptitude^63^). The optical density of the bacterial solution was 1.5, and the concentration of urea and calcium chloride was 1 M (selected based on the values suggested in the previous studies^36,64,65^). The culture media comprised nutrient broth (8 g/L) together with urea (20 g/L). The bacterial solution was sprayed on the dune surfaces and they were left for 24 h for bacterial attachment. After 24 h of the attachment period, the cementation solution (calcium chloride and urea) was sprayed. The ureolytic MICP control tests are denoted as UMC hereafter. The calcium carbonate content of soil samples treated with ureolytic and non-ureolytic pathways was obtained by means of the washing method following the procedure suggested by Choi et al.^66^.

## Results

### Effect of initial culture medium pH on growth of bacteria

**Figure 5:**
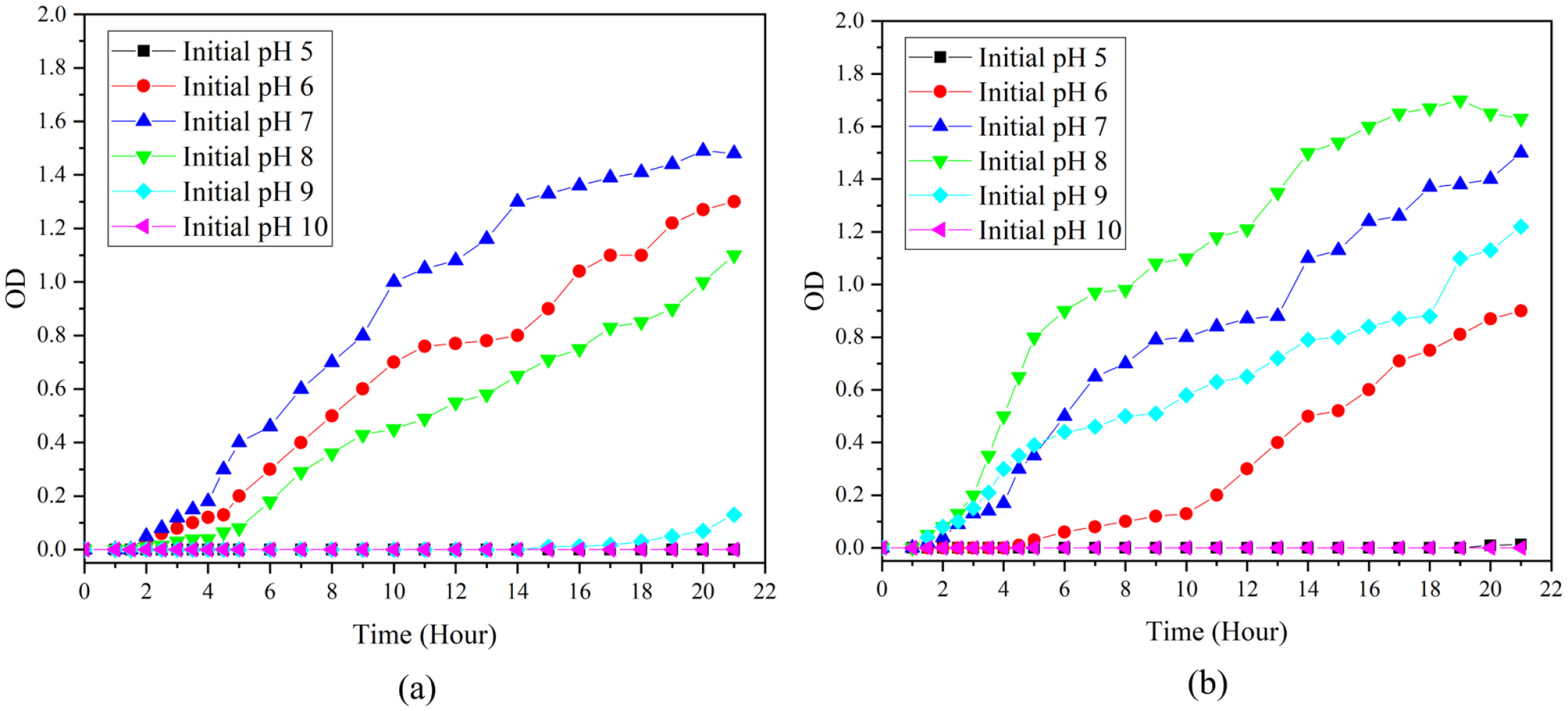
Growth curves of (**a**) Bacillus amyloliquefaciens (**b**) Bacillus subtilis, at different initial culture medium pH values.

Figure 5 presents the growth curve of bacteria for bacillus amyloliquefaciens, and bacillus subtilis in the culture media (Nutrient broth), with the initial pH ranging from 5 to 10. As the figures reveal, the faster growth of bacillus amyloliquefaciens, and bacillus subtilis has occurred in pHs 6–8 and 7–9, respectively. Therefore, this pH range has been employed in the optimization stage.

### Optimization of calcium carbonate production

**Figure 6:**
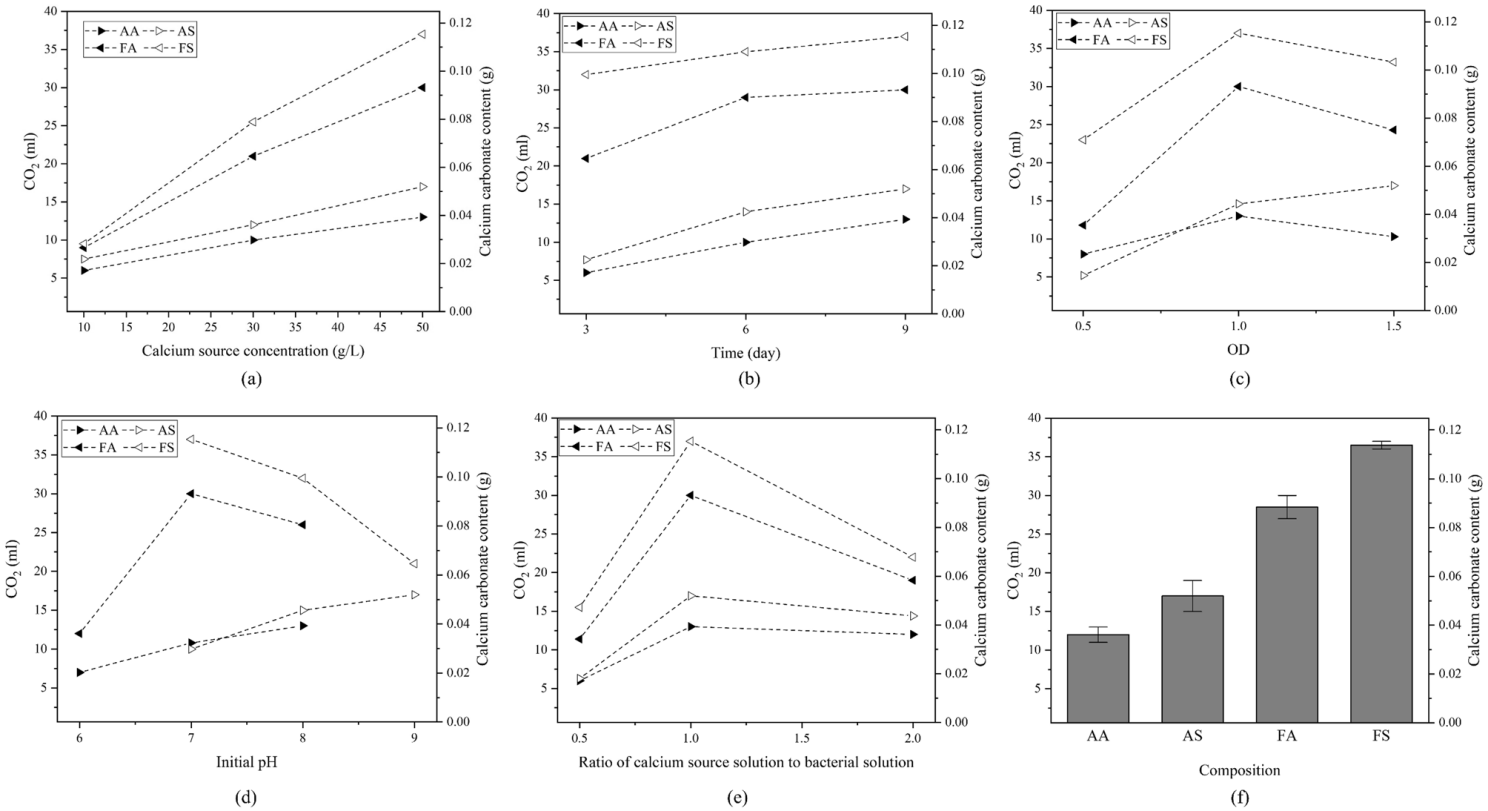
The variation of CO_2_ volume measured in Bernard calcimeter (as well as corresponding CaCO_3_ content) with (**a**) calcium source concentration, (**b**) curing time, (**c**) OD, (**d**) initial pH, (**e**) ratio of calcium source to bacterial solution, for each composition; and (**f**) maximum amount of calcium carbonate produced for each calcium source-bacteria composition.

Figure 6 presents the amount of CO_2_ produced in the Bernard calcimeter, which is a proxy for the precipitated CaCO_3_. Since for each combination, one factor is fixed and others are varied, each point in these figures corresponds to the maximum amount of CO_2_ volume for the set of experiments. As the diagrams reveal, CaCO_3_ production increased by increasing the concentration of calcium sources. Therefore, calcium source concentration directly affects calcium carbonate production. As calcium and carbon sources are the same (i.e., calcium formate and calcium acetate), the more calcium ions are released, the more CaCO_3_ is produced (Fig. 6a). In compositions of AS and AA, by increasing the curing time, the amount of produced calcium carbonate increased incessantly until 9 days after which there was little change in precipitation amount. Whereas, in FA composition, the rate of CaCO_3_ production was reduced with times surpassing six days. The FS composition experienced a relatively low rate of CaCO_3_ production right after three days as compared to other compositions (Fig. 6b). In the FA and FS compositions, 70% and 87% of total calcium carbonate production has been produced just after three days, while in AA and AS compositions, it is only around 46% and 45%, respectively. This indicates a faster CaCO_3_ production in the formated-based compositions at the beginning compared to the acetate-based compositions. Nevertheless, the rate slows down with the increase in curing time. From Fig. 6c, it may be concluded that an increase in the bacterial concentration beyond the OD of one does not have an appreciable contribution to the produced calcium carbonate.

As for the effect of the initial pH of the culture medium, Fig. 6d shows that for the FA and FS, the maximum CaCO_3_ production occurs at the pH of 7. This observation agrees well with past studies stating that the FDH enzyme was most stable at the pH of 7^67^. However, for AA and AS, some increase in CaCO_3_ precipitation was observed for pHs beyond 7. Previous studies also indicate that the optimum pH range for CoA enzyme activity is 8 to 9.2^68^. Considering the optimum pH ranges for CoA enzyme activity and bacillus amyloliqufaciens growth of (8–9.2) and (6–8) (Fig. 5a), respectively, the expected optimum pH for AA composition would be 8, where the two pH intervals overlap. This fact was exrimentally proved, as shown in Fig. 6d. As the optimum pH interval for the bacillus subtilis growth is 7 to 9 (Fig. 5b), and given the optimum pH for CoA enzyme activity, namely, 8 to 9.2, the maximum CaCO_3_ precipitation is anticipated to be located in the pH range of 8–9 which is confirmed by Fig. 6d (i.e., the optimum precipitation pH is determined to be 9). Results shown in Fig. 6e indicate that the optimum ratio of calcium source solution to bacterial solution is equal to one for both acetate- and formate-based solutions. For the sake of comparison, the maximum amount of CaCO_3_ production at different conditions (i.e., calcium source concentration, curing time, OD, the ratio of calcium source to the bacterial solution, and initial pH) was employed as an index to assess the performance of different compositions, namely, AA, AS, FA and, FS. The maximum CaCO_3_ production among the studied compositions belongs to the FS group, which produces CaCO_3_ approximately three times the AA composition (Fig. 6f). Four control experiments were performed without bacteria for both calcium sources, and after 30 days, no CaCO_3_ precipitate was observed.

#### Crystallographic study of biologically precipitated calcium carbonate

**Figure 7:**
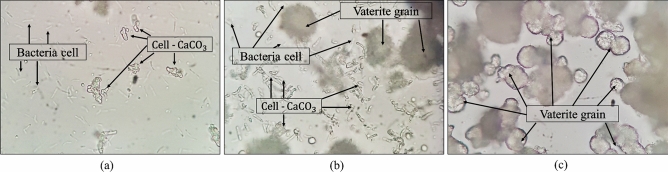
The optical microscope observation of the CaCO_3_ precipitation with time for the FS composition: after (**a**) 24, (**b**) 48, and (**c**) 72 h.

Photomicrographs of all compositions obtained by optical microscope indicate that vaterite is the dominant phase for CaCO_3_ production (Fig. 7). The vaterite crystals take spherical forms^69–71^. It was observed that calcium carbonates were precipitated on the bacteria cells because the surfaces of bacterial cells are negatively charged and act as adsorbents of divalent cations. In the current study, for instance, for the FS composition and after 24 h, calcium carbonate commenced to form on some bacteria cells (Fig. 7a), and after 48 h, the number of bacteria cells covered with the calcium carbonate increased considerably. Moreover, vaterite particles were also detectable, as shown in Fig. 7b. Finally, after 72 h, a significant number of the bacteria appeared confined by the vaterite crystals, and the number of vaterite particles increased significantly (Fig. 7c).

**Figure 8:**
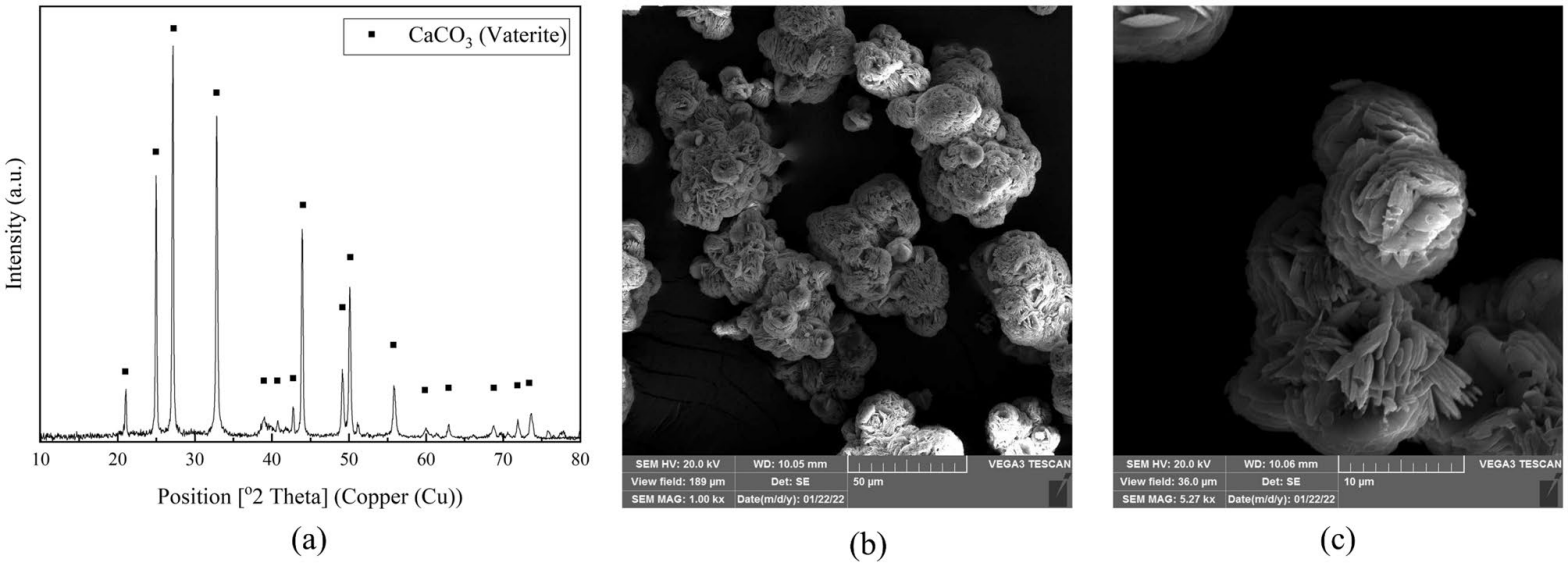
(**a**) XRD spectrum for produced CaCO_3_ as compared to vaterite. SEM micrograph of vaterite at magnification of (**b**) 1 kx, and (**c**) 5.27 kx.

XRD and SEM analyses were performed on the powder to inspect the form of the precipitated phase further. The XRD spectrum (Fig. 8a) and the SEM micrographs (Fig. 8b,c) confirm the presence of vaterite crystals due to their lettuce-like shape as well as the agreement between peaks of vaterite and the precipitated material.

### Wind tunnel test results

#### TDV and wind erosion of sand dune

**Figure 9:**
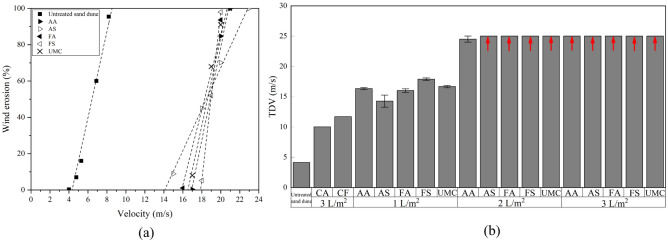
Wind erosion tests (**a**) weight loss versus wind velocity (application rate of 1 L/m^2^), (**b**) threshold detachment velocity versus application rate and compositions (CA stands for calcium acetate, and CF for calcium formate).

The wind tunnel test results are presented in Fig. 9a,b. As clear from Fig. 9a, the threshold detachment velocity (TDV) of the untreated sand was determined to be approximately equal to 4.32 m/s. At the application rate of 1 L/m^2^, Fig. 9a, the slopes of the soil loss-velocity line for FA, FS, and AA compositions as well as UMC are approximately equal to that of the untreated sand dunes. This demonstrates that the treatments at this application rate are ineffective, and as soon as the wind velocity surpasses the TDVs, the thin soil crusts would vanish and erosion of the dune takes place at the same rate as in the untreated case. The erosion slope for the AS composition was lower than other compositions with a lower abscissa (i.e., TDV) as well (Fig. 9a). Arrows in Fig. 9b indicate that in the treated sand dunes with application rates of 2 and 3 L/m^2^, erosion did not occur at the maximum wind velocity of 25 m/s. In other words, for FS, FA, AS, and UMC at application rates of 2 and 3 L/m^2^, resistance to the wind erosion of sand dunes caused by CaCO_3_ precipitation was higher than the maximum wind velocity (i.e., 25 m/s). Hence, the reported TDV value of 25 m/s in these tests is a lower limit for the application rates shown in Fig. 9b, except for case AA, where TDV is almost equal to the maximum speed of the wind tunnel.

**Figure 10:**
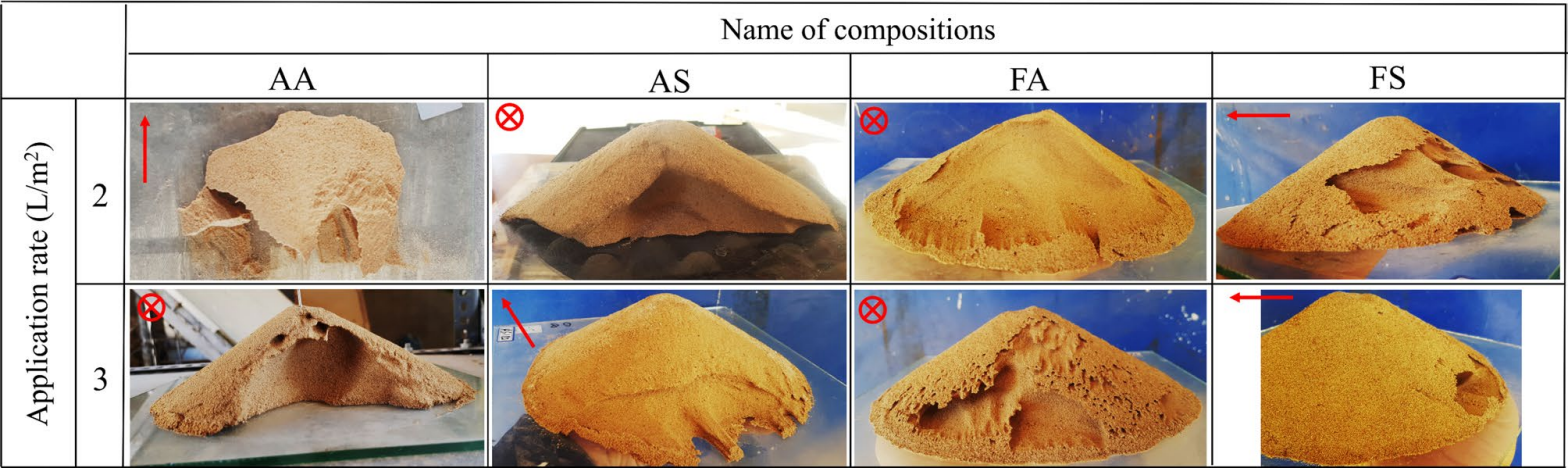
Treated sand dunes after sand bombardment for different compositions at 2 and 3 L/m^2^ application rate (the arrows indicate wind direction, and the cross denotes wind direction perpendicular to the figure plane).

**Figure 11:**
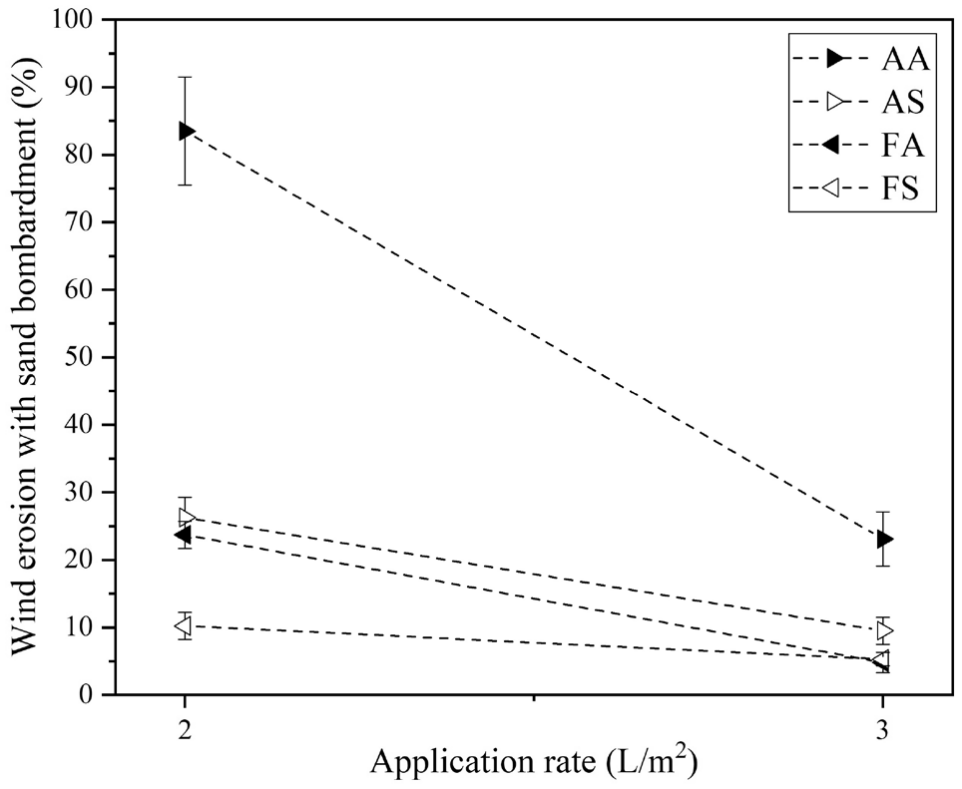
Wind erosion after sand bombardment versus application rate.

Figure 10 shows surface erosion of treated dunes after sand bombardment tests for different compositions and application rates, and quantitative results are shown in Fig. 11. The untreated case is not presented as it did not show any resistance and was totally eroded (total mass loss) during the sand bombardment test. As clear from Fig. 11, and for the applicate rate of 2 L/m^2^, the sample treated with AA biological compound experienced 83.5% weight loss, while all other samples experienced less than 30% erosion during the sand bombardment. By increasing the application rate to 3 L/m^2^, all treated samples underwent less than 25% weight loss. The best performance against sand bombardment for both application rates belonged to the FS compound. The maximum and minimum bombardment resistance among treated samples in FS and AA compounds could be attributed to their maximum and minimum CaCO_3_ precipitation (Fig. 6f).

#### Results of calcium carbonate content and surface resistance

**Figure 12:**
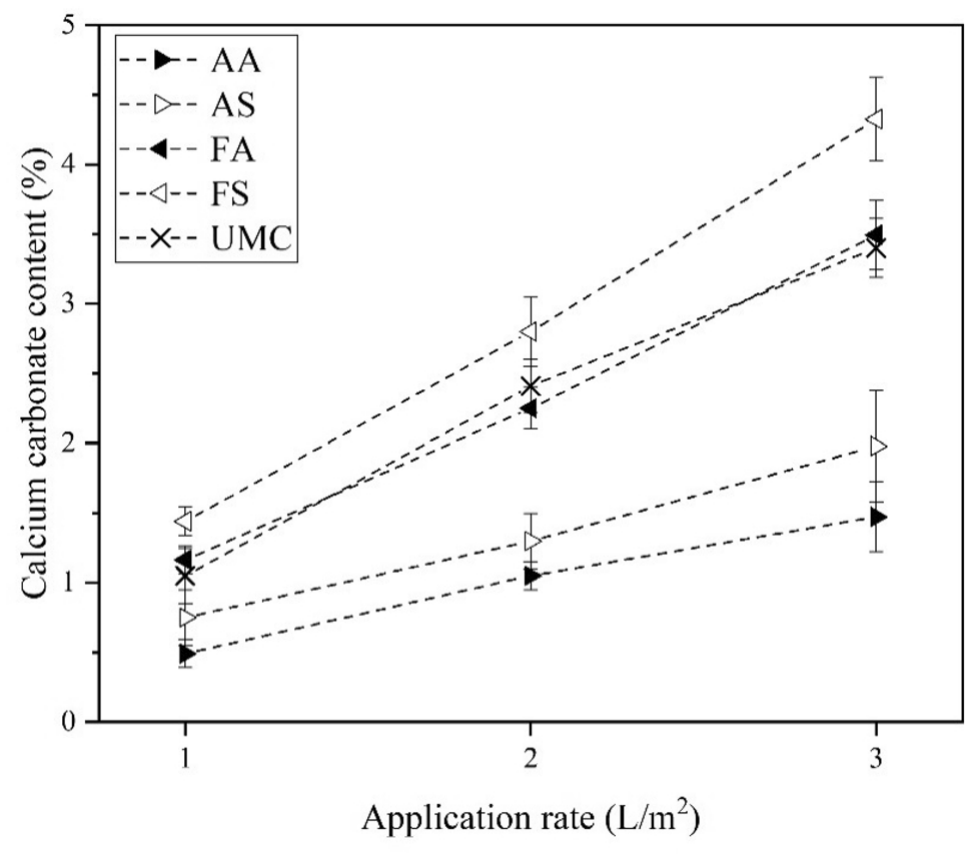
Calcium carbonate content versus application rate.

As can be seen in Fig. 12, by increasing the application rate from 1 L/m^2^ to 3 L/m^2^, the calcium carbonate content also increases for all compositions. Furthermore, the composition with the highest calcium carbonate content is FS at all application rates, followed by FA together with UMC. This suggests that these compositions may have higher surface resistance.

**Figure 13:**
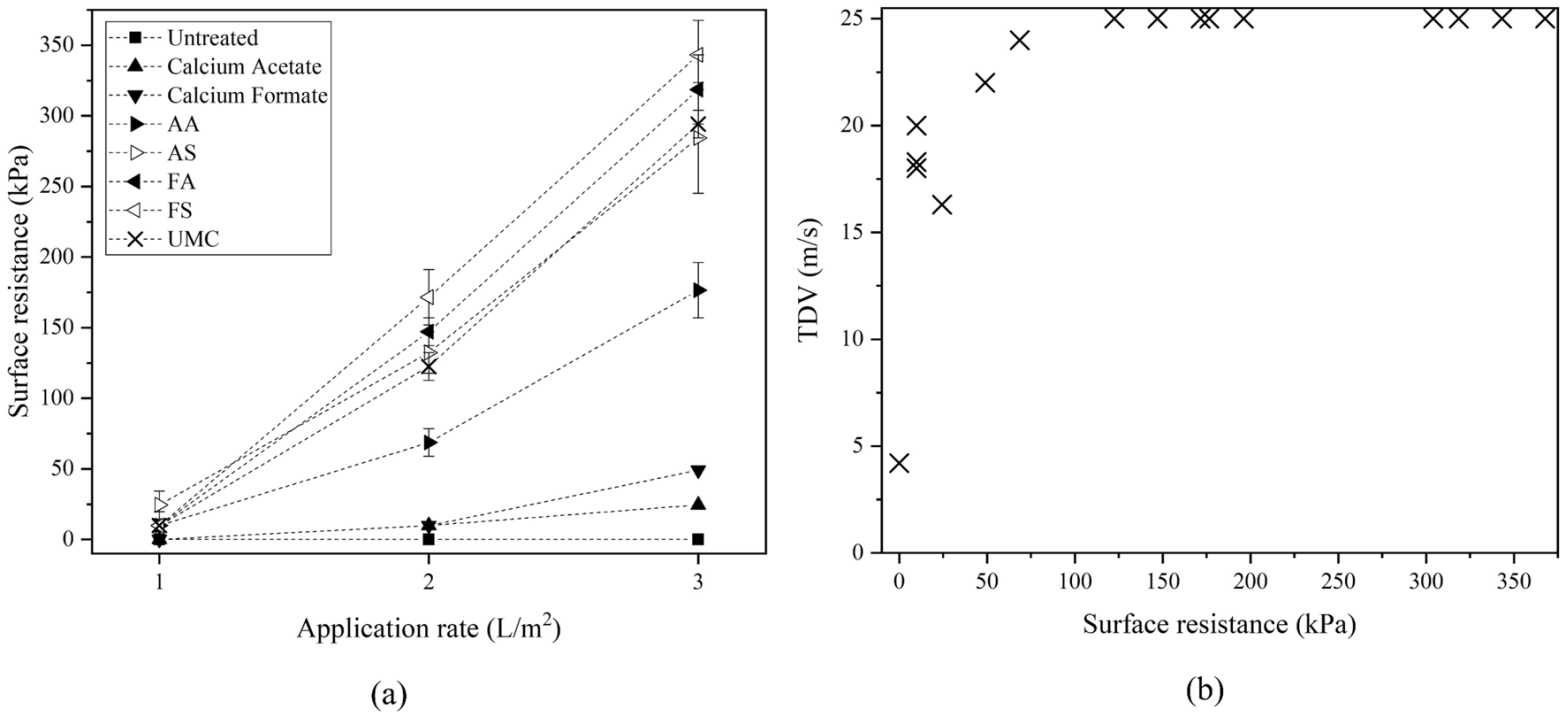
(**a**) Surface resistance versus application rate. (**b**) TDV versus surface resistance.

Figure 13a shows the change in surface resistance measured from penetrometer tests for untreated, control, and treated soil samples. From this figure, it may be concluded that the surface resistance in UMC, AS, FA, and FS compositions increases significantly with the increase in the application rate. However, this increase in surface strength is relatively less pronounced in the AA composition. As revealed by this figure, FA and FS compositions of non-ureolytic MICP resulted in better surface penetration as compared to ureolytic MICP. Figure 13b illustrates the variation in TDV as a function of soil surface resistance. From this figure, it is clear that for sand dunes with the surface resistance of over 100 kPa, the threshold detachment speed will exceed 25 m/s. As the in-situ surface resistance can be easily measured using a penetrometer device, such a piece of knowledge can help to estimate TDV in the absence of a wind tunnel test and thereby serve as a quality control index for field applications.

#### Results of SEM

**Figure 14:**
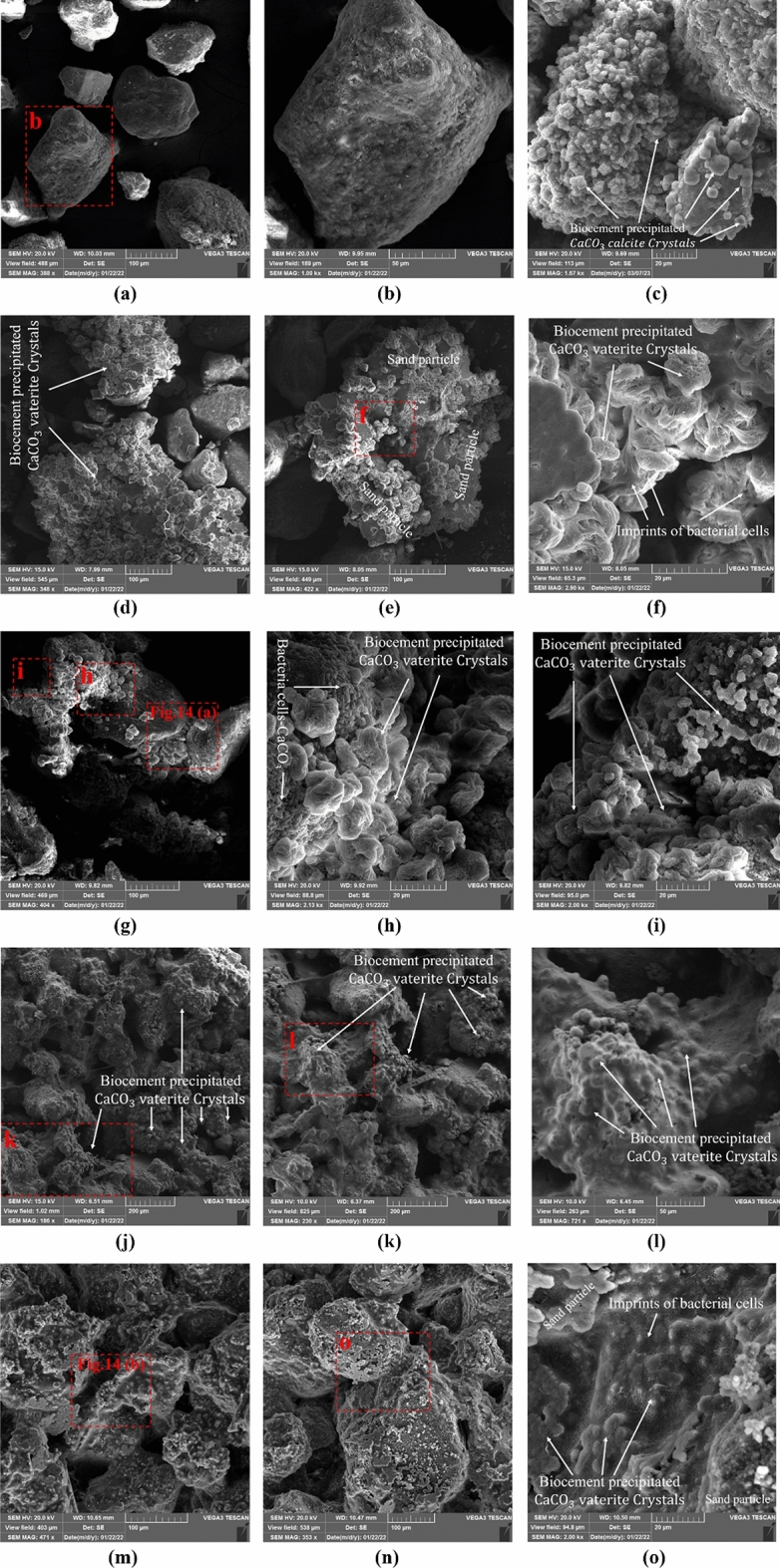
SEM micrograph of (**a**, **b**) untreated soil, (**c**) control ureolytic MICP, (**d**-**f**) AA-composition treated sample, (**g**–**i**) AS-composition treated sample, (**j**–**l**) FA-composition treated sample, (**m**–**o**) FS-composition treated sample, at application rate of 3 L/m^2^ at different magnifications.

The results of SEM are displayed in Fig. 14. Figure 14a–b show the magnified particles of the untreated soil sample, clearly indicating its cohesionless nature and absence of natural bond or cementation. Figure 14c presents the SEM micrograph of control sample treated with ureolytic MICP. The figure indicates the presence of CaCO_3_ precipitations as calcite polymorph. As shown in Fig. 14d–o, the precipitated CaCO_3_ has bonded the grains together; spherical vaterite crystals are also identifiable in the SEM photomicrographs. The results of this study along with previous ones indicate that the CaCO_3_ bonds formed as vaterite polymorph can also provide reasonable mechanical strength; our results indicated an improvement in surface resistance up to 350 kPa as well as an increase in the threshold detachment velocity from 4.32 to more than 25 m/s. The results agree with the findings of previous studies, where precipitated CaCO_3_ substrate induced by MICP was vaterite, in which reasonable mechanical strength and wind erosion resistance was achieved^13,40^ and reasonable wind erosion resistance was maintained even after 180 days exposed to field environmental conditions^13^.

Figure 14d–f reveal that treatment with the AA compound has resulted in the precipitation of calcium carbonate on and among the sand grains, while some uncovered grains were also noticed. For the AS compositions, although the amount of produced CaCO_3_ has not increased substantially (Fig. 6f), the number of CaCO_3_-induced contacts among the sand grains has increased significantly compared to the AA compound (Fig. 14 g–i).

From Fig. 14j–l and 14m–o, it is clear that the use of calcium formate as the calcium source results in a further increase of CaCO_3_ precipitation compared to the AS compound, which is in agreement with calcimeter measurements indicated in Fig. 6f. It seems that this additional CaCO_3_ has mainly been precipitated on the sand grains and may not necessarily boost the contact quality. This can justify the previously observed behavior that despite the difference in the amount of CaCO_3_ precipitation (Fig. 6f), the resistance to wind erosion with sand bombardment (Fig. 11), and surface resistance (Fig. 13a) of all these three compositions, namely, AS, FA, and FS, are not that different.

**Figure 15:**
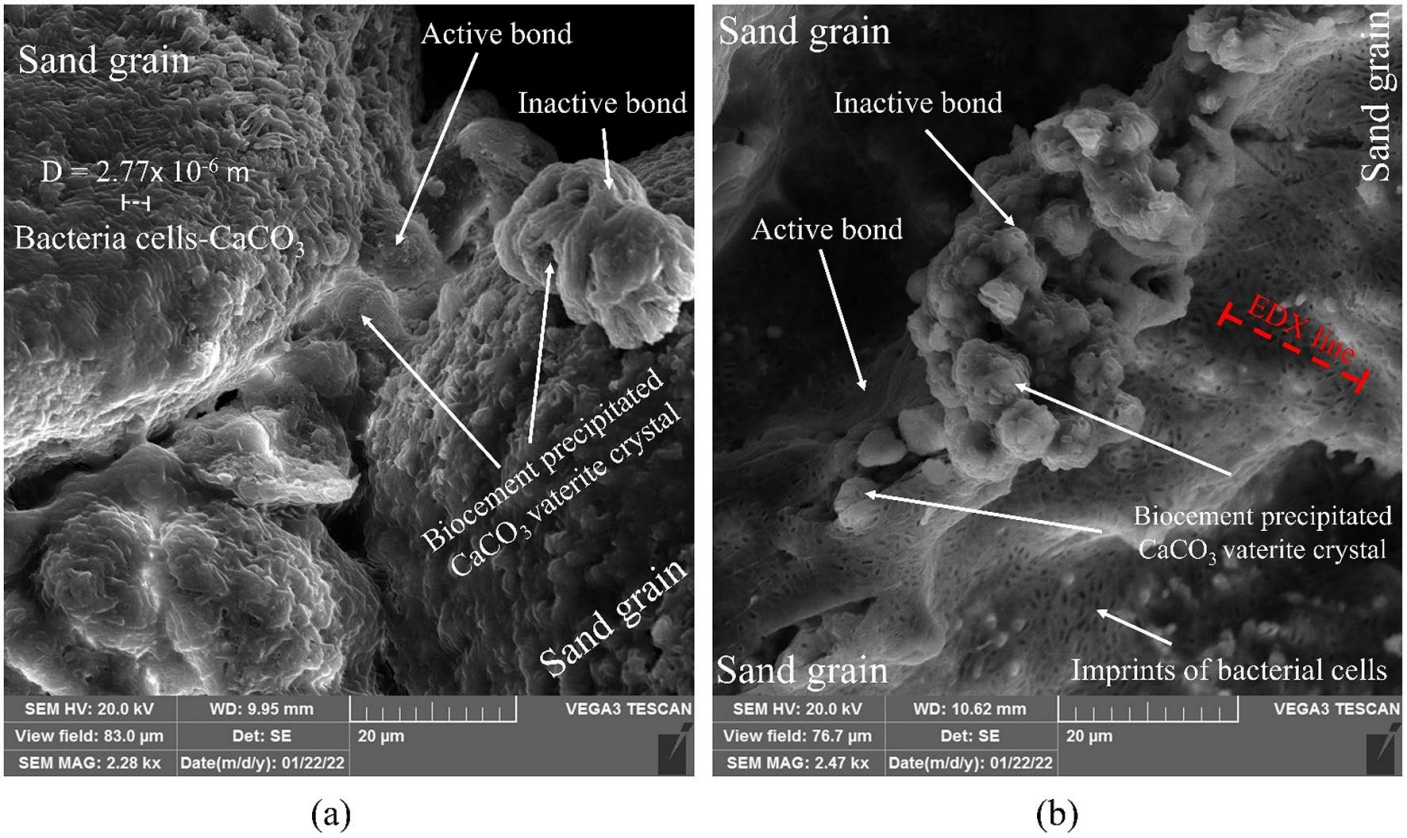
CaCO_3_ induced active and inactive bonds, and imprints of bacteria cells on precipitation in (a) AS-composition treated sample, and (b) FS-composition treated sample.

In order to better observe the presence of bacteria cells covered by CaCO_3_ as well as the imprint of bacteria on the precipitated crystals, SEM micrographs of higher magnification were employed, and the results are presented in Fig. 15. As the figure discloses, calcium carbonate precipitates on the bacteria cells, where they provide the required nuclei for precipitation. This figure also depicts active and inactive bonds induced by CaCO_3_. It may be deduced that any increase in the inactive bonds does not necessarily provide further improvement in the mechanical behavior. Thus, the increase in CaCO_3_ precipitation does not necessarily result in higher mechanical strength, and the precipitation pattern plays an important role. This point has also been addressed in the works of Terzis and Laloui^72^ and Soga and Al Qabany^45,73^. To further dig into the relationship between the precipitation pattern and mechanical strength, the use of micro-CT imaging studies is recommended, which was outside the scope of the current study (i.e., the introduction of different calcium source-bacterial compositions for ammonia-free MICP).

**Figure 16:**
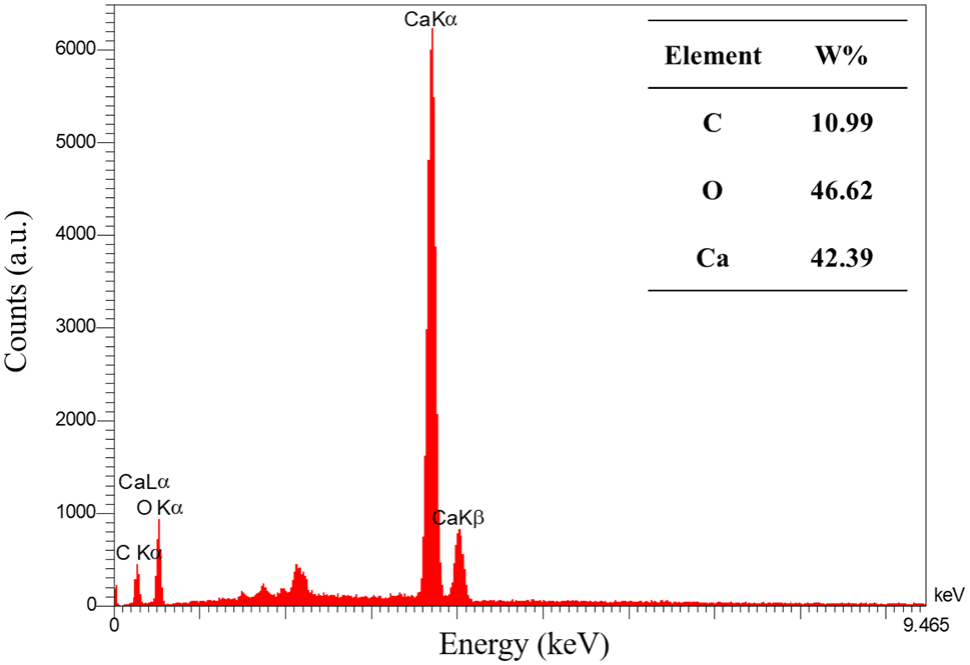
EDX analysis of the film covering the grains in the samples treated with formate-based compositions.

As indicated in Figs. 14j–o and Fig. 15b, there is a thin film of CaCO_3_ (according to EDX analysis, the percentage compositions of each element in the thin film are carbon 11%, oxygen 46.62%, and calcium 42.39%, which is very close to the elemental percentage of CaCO_3_ (Fig. 16)). The film covers the vaterite crystal and soil grains, contributing to the integrity of the soil-precipitation system. The presence of this film was solely observed in the samples treated with formate-based compositions.

## Ureolytic versus non-ureolytic MICP for wind erosion mitigation

A comparison of obtained surface strength, threshold detachment velocity, and biologically induced CaCO_3_ content of the treated soils reported in previous studies and those of the current study using both ureolytic and non-ureolytic MICP pathways is presented in Table2. There are limited studies on the wind erosion resistance of MICP-treated dune samples. Meng et al. studied wind erosion resistance of ureolytic MICP-treated sand dune samples using a wind blower^13^, whereas in the current study, the non-ureolytic (as well as control ureolytic) treated dune samples were examined in the wind tunnel and treatment was performed with four different bacteria-substance compositions.

**Table 2:** Ureolytic versus non-ureolytic MICP for wind erosion mitigation.

Study no	MICP pathway	Bacteria	Calcium source	TDV (m/s)	Application rate (L/m^2^) (MICP cycle)	Surface resistance value (kPa) (Calcium carbonate content (%))	Surface shape	References
1	Ureolytic	Pseudogracilibacillus auburnensis	Calcium chloride	15.3	1.62 (1)	NA^*^ (1.75)	Flat	Dubey et al.^65^
2	Ureolytic	Bacillus megaterium	Calcium chloride	12.5	1.62 (1)	NA (2.97)	Flat	Devrani et al.^76^
3	Ureolytic	Sporosarcina pasteurii	Calcium chloride	30	4 (1)	300 (0.57)	Dune	Meng et al.^13^
4	Ureolytic	Sporosarcina pasteurii	Calcium chloride	16	5 (3)	NA (NA)	Flat	Tian et al.^74^
5	Ureolytic	Sporosarcina pasteurii	Calcium chloride	+ 25	2 (1)	132 (2.41)	Dune	Current study (Control test)
6	Non-ureolytic	Bacillus megaterium	Calcium acetate	+ 25	4 (1)	NA (2.6)	Flat	Fattahi et al.^41^
7	Non-ureolytic	Bacillus amyloliquefaciens	Calcium acetate	30	2 (1)	NA (NA)	Flat	Mohebbi et al.^9^
8	Non-ureolytic	Bacillus subtilis	Calcium formate	+ 25	2 (1)	190 (2.8)	Dune	Current study
9	Non-ureolytic	Bacillus amyloliquefaciens	Calcium formate	+ 25	2 (1)	157 (2.25)	Dune	Current study
10	Non-ureolytic	Bacillus subtilis	Calcium acetate	+ 25	2 (1)	150 (1.3)	Dune	Current study
11	Non-ureolytic	Bacillus amyloliquefaciens	Calcium acetate	24	2 (1)	78.44 (1.05)	Dune	Current study

*NA: the values were not reported.

As can be seen, some of the previous studies have considered high application rates, more than 4 L/m^2^^13,41,74^. It is worth mentioning that high application rates may not be easily applicable to the field when regarded from an economic standpoint due to the costs associated with water supply, transportation, and application of significant amounts of water. Lower application rates as low as 1.62–2 L/m^2^ have also achieved reasonably well surface strength as high as 190 kPa, and TDV exceeding 25 m/s. In the current study, format-based non-ureolytic MICP treated dunes gained high surface strength comparable to the values gained in the ureolytic pathway in the same range of application rate (i.e., formate-based non-ureolytic MICP treated samples at higher application rates could also reach surface strength values in the same range of surface strength reported by Meng et al.^13^, Fig. 13a). Furthermore, it can be seen that at the application rate of 2 L/m^2^, and for the formate-based non-ureolytic MICP, the amount of produced calcium carbonate which mitigated the wind erosion at 25 m/s is 2.25%, when compared with dunes treated with the control ureolytic MICP at the same application rate and subjected to the same wind speed (25 m/s), the required CaCO_3_ amount was found to be very close (i.e., 2.41%).

Thus, it can be concluded from this table that both ureolytic and non-ureolytic pathways result in fairly reasonable performance in terms of surface resistance and TDV. The major difference is that non-ureolytic pathway is ammonia-free and will, thereby, lead to less environmental concerns. Furthermore, the formate-based non-ureolytic MICP proposed in this study seems to perform better than the acetate-based non-ureolytic MICP. Although Mohebbi et al. investigated acetate-based non-ureolytic MICP, their study concerned samples of flat surface^9^. Wind erosion of the dune samples is expected to be much more pronounced than the flat surface at the same velocity due to the formation of an eddy current around dune samples and its induced shear, which leads to higher erosion, and thereby, lower TDV^75^.

## Conclusion

Due to its low greenhouse gas footprint, microbial-induced carbonate precipitation (MICP) is an eco-friendly soil improvement technique. However, ureolytic MICP by-products, such as ammonia from urea hydrolysis, are unfavorable. To address this issue, this study explored carbonate precipitation using non-ureolytic MICP and comprehensively examined its application for wind erosion mitigation. Two new compositions for non-ureolytic MICP, namely, calcium formate-bacillus subtilis and calcium formate-bacillus amyloliquefaciens (FS and FA) were studied. Their performance for calcium carbonate production and erosion mitigation in sand dunes were examined against acetate-based compositions (calcium acetate-bacillus subtilis and calcium acetate-bacillus amyloliquefaciens, AS and AA) as well as a control ureolytic MICP by Sporosarcina pasteurii.

The optimized values of factors controlling CaCO_3_ precipitation were obtained for the four bacteria-calcium source compositions. Factors included calcium source concentration, curing time, OD, the ratio of calcium source to bacterial solution, and pH. Calcium formate resulted in higher CaCO_3_ production than calcium acetate, and bacillus subtilis was slightly better than bacillus amyloliquefaciens. FS was the best compound for producing calcium carbonate. Compositions with calcium formate had shorter curing times. The produced calcium carbonate phase was identified as spherical lettuce-like vaterite.

The optimized factors were applied to sand dune samples for wind tunnel experiments at three application rates (1, 2, and 3 L/m^2^). At application rate of 2 L/m^2^, all compositions except for AA had a TDV higher than 25 m/s. In sand bombardment experiments, samples treated with all compositions except for AA at an application rate of 2 L/m^2^ experienced less than 30% weight loss, and at 3 L/m^2^, all treated samples suffered less than 25% weight loss. The FS and AA compounds had maximum and minimum bombardment resistance. Increasing the application rate substantially increased the surface resistance of samples treated with AS, FA, and FS, while the AA composition performed poorly.

Comparing non-ureolytic MICP wind erosion mitigation of sand dunes with control ureolytic MICP indicated that formate-based compositions resulted in surface strength and wind erosion resistance performance competing with the standard ureolytic MICP pathway, while they do not suffer from the adverse environmental effects of ammonia by-products. In addition, CaCO_3_ content measurements of treated samples indicated that the composition with the highest calcium carbonate content was FS, followed by FA and the control ureolytic MICP test.

SEM and XRD results confirmed vaterite formation bonding the grains together and covering their surface, while less precipitation was observed in AA-treated samples. Bacteria cells were found to play an essential role as precipitation nuclei. The difference in surface resistance by FS and FA compositions is less pronounced than their CaCO_3_ precipitation difference, suggesting the precipitation pattern also plays a role, which requires further study through micro-CT imaging.

Finally, applying biological CaCO_3_ precipitation through organic material oxidation by heterotrophic bacteria could be the leading edge for other soil improvement applications. Hence, future studies are encouraged to use the proposed calcium source-bacteria compositions to improve different soil hydraulic and mechanical properties.

## Data Availability

Data would be available upon request from the corresponding author.
